# Extremely brilliant GeV γ-rays from a two-stage laser-plasma accelerator

**DOI:** 10.1126/sciadv.aaz7240

**Published:** 2020-05-29

**Authors:** Xing-Long Zhu, Min Chen, Su-Ming Weng, Tong-Pu Yu, Wei-Min Wang, Feng He, Zheng-Ming Sheng, Paul McKenna, Dino A. Jaroszynski, Jie Zhang

**Affiliations:** 1 Key Laboratory for Laser Plasmas (MOE), School of Physics and Astronomy, Shanghai Jiao Tong University, Shanghai 200240, China.; 2 SUPA, Department of Physics, University of Strathclyde, Glasgow G4 0NG, UK.; 3 Collaborative Innovation Center for IFSA, Shanghai Jiao Tong University, Shanghai 200240, China.; 4 Department of Physics, National University of Defense Technology, Changsha 410073, China.; 5 Department of Physics, Renmin University of China, Beijing 100872, China.; 6 Institute of Physics, Chinese Academy of Sciences, Beijing 100190, China.; 7 Cockcroft Institute, Sci-Tech Daresbury, Cheshire WA4 4AD, UK.; 8 Tsung-Dao Lee Institute, Shanghai Jiao Tong University, Shanghai 200240, China.

## Abstract

Recent developments in laser-wakefield accelerators have led to compact ultrashort X/γ-ray sources that can deliver peak brilliance comparable with conventional synchrotron sources. Such sources normally have low efficiencies and are limited to 10^7–8^ photons/shot in the keV to MeV range. We present a novel scheme to efficiently produce collimated ultrabright γ-ray beams with photon energies tunable up to GeV by focusing a multi-petawatt laser pulse into a two-stage wakefield accelerator. This high-intensity laser enables efficient generation of a multi-GeV electron beam with a high density and tens-nC charge in the first stage. Subsequently, both the laser and electron beams enter into a higher-density plasma region in the second stage. Numerical simulations demonstrate that more than 10^12^ γ-ray photons/shot are produced with energy conversion efficiency above 10% for photons above 1 MeV, and the peak brilliance is above 10^26^ photons s^−1^ mm^−2^ mrad^−2^ per 0.1% bandwidth at 1 MeV. This offers new opportunities for both fundamental and applied research.

## Teaser

A novel scheme of staged laser-plasma accelerators produces extremely brilliant γ-rays with photon energies up to GeV level.

## INTRODUCTION

Bright sources of high-energy γ-rays are versatile tools (*1*–*3*) that are applied in broad areas ranging from fundamental research (*4*–*7*) in astrophysics, particle and nuclear physics, to high-resolution imaging (*8*, *9*) in chemistry, biology, medicine, materials science, and industry. These applications can benefit greatly from the availability of further compact γ-ray sources with low divergence, short pulse duration, high energy, and high peak brilliance. At present, widely used synchrotrons (*2*) and X-ray free-electron lasers (XFELs) (*10*) can deliver X-ray pulses with peak brilliance in the range of 10^19–24^ and 10^27–32^ photons s^−1^ mm^−2^ mrad^−2^ per 0.1% bandwidth (BW), respectively. However, they are normally limited to photon energies ranging from a few keV to hundreds of keV, and peak powers in the multi-GW level. In addition, the size and cost of these large research infrastructures limit access to the sources.

On the other hand, compact laser-wakefield accelerators (LWFAs) (*11*–*16*) have been developed rapidly over the past two decades (*17*) and offer a radically different approach—the acceleration length in plasmas is about three orders of magnitude smaller as compared to conventional accelerators, providing the ability to drive the acceleration and radiation of high-energy particles on a much smaller scale. Multi-GeV electron beams have been produced using LWFA (*18*), and femtosecond-scale X/γ-ray pulses in the range of keV to MeV can be produced via LWFA-based betatron radiation (*19*–*22*) and Compton backscattering (*23*–*25*). The resulting radiation sources have typical peak brilliance of 10^19–23^ photons s^−1^ mm^−2^ mrad^−2^ per 0.1% BW, while the photon number per shot is limited to 10^7–8^ photons with the laser-to-photon energy conversion efficiency at a very low level on the order of 10^−6^. Although substantial efforts have been dedicated to enhancing betatron radiation, such as using energetic particle beam–driven plasma wakefields (*26*, *27*) and increasing transverse oscillation amplitudes (*28*), it remains a great challenge to significantly increase the energy conversion efficiency and to generate collimated γ-rays with high peak brilliance on the order of the XFEL level and with high energies in the MeV to GeV regime. Currently, many cutting-edge applications and scientific research (*29*–*31*) require γ-rays with ultrahigh brilliance and photon energies far exceeding 1 MeV. These applications include exploring elementary particles (*4*), probing nuclear structures and photonuclear physics (*5*, *6*), and examining quantum processes (*7*), which rely heavily on γ-ray sources in the MeV to GeV range.

Continuous development in ultrahigh-power laser technology (*32*) provides possibilities for producing brilliant high-energy γ-ray sources. So far, considerable theory and simulation efforts have been made to develop such photon sources, based on emission of energetic electrons accelerated in extreme laser fields, such as laser interactions with near-critical-density plasmas (*33*–*35*), laser-driven radiation reactions (*36*–*38*), laser-irradiated solid interactions (*39*–*41*), laser scattering off electrons (*42*, *43*), and the excitation of electromagnetic cascades (*44*, *45*). However, there are unavoidable physical limitations on the γ-ray peak brilliance with these methods, such as a very large divergence in direct laser interaction with electrons. Furthermore, an exceptionally high laser intensity of 10^23–25^ W/cm^2^ (two to four orders of magnitude higher than the highest intensities available to date) is required to produce GeV photons. This requires tens-of-petawatt (PW) laser pulses to be focused to near-diffraction-limited spots, which is very challenging. As soon as the laser intensity is reduced to the order of ~10^21^ W/cm^2^ (which is the highest intensity level of reliable operation of current high-power laser systems), the methods mentioned above become intrinsically inefficient for γ-ray emission. In addition, the ability to tune the photon energy, power, and brilliance is limited. It has been recently proposed that collimated γ-rays may be produced when ultradense relativistic electrons interact with conductors via beam-plasma instabilities (*46*), but the required high-density GeV electron beams are well beyond current technical capabilities. Heretofore, there is no alternative method applicable to achieve the peak brilliance of γ-ray sources comparable to the XFEL level.

Here, we introduce a new efficient scheme to produce extremely high-brilliance γ-rays with photon energies up to GeV, which is based on a two-stage LWFA driven by a single multi-PW laser pulse. The first stage, using a moderately low-density plasma, produces a multi-GeV electron beam with a high energy efficiency of ~40%, while the second stage using a relatively high-density plasma produces MeV-GeV γ-ray radiation with an efficiency over 10%. The resulting photon number, energy efficiency, peak brilliance, and power are several orders of magnitude higher than existing LWFA-based sources. This may pave the way for applications in broad areas of science and technology that require high-brilliance γ-rays with photon energy in the MeV to GeV regime.

## RESULTS

### Physical scheme

It is well known that a low-density plasma is beneficial for accelerating trapped electrons to high energies because the electron dephasing length (*17*) *L*_deph_ ∝ 1/*n_e_*, where *n_e_* is the plasma density. However, strong betatron oscillations preferentially occur in a high-density plasma, which enhances betatron radiation with a critical photon energy $\hbar \omega_{\text{crit}}\propto {\left(\frac{\epsilon_{e}}{m_{e}c^{2}}\right)}^{2}n_{e}r_{\beta }$, where *c* is the speed of light in vacuum and ϵ_e_, *m_e_*, and *r*_β_ are the electron energy, mass, and transverse oscillation amplitude, respectively. This contradiction severely restricts betatron emission in the wakefield to photon numbers in the range 10^7–8^ and photon energy in the hundreds of keV range, thus only achieving peak brilliance comparable to the synchrotron radiation.

**Fig. 1:**
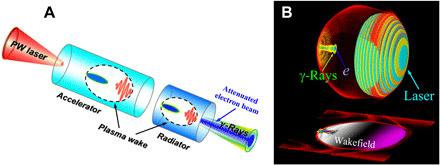
Concept of the compact bright γ-ray source. (**A**) Schematic of the two-stage scheme. In the first acceleration stage, a plasma wake is driven by a multi-PW laser pulse propagating in an underdense plasma channel, where efficient electron injection and acceleration result in a multi-GeV, low-emittance, high-charge, and high-density electron beam. The laser pulse then enters a higher-density plasma region that acts as a radiator, where collimated bright γ-rays are produced by the dense high-energy electrons in the enhanced electrostatic fields of the bubble in the denser plasma. (**B**) Three-dimensional (3D) view of the γ-ray radiation in laser-driven plasma wakefield using a 3D particle-in-cell (PIC) simulation. Simulation parameters are mentioned in Methods.

To overcome this problem, we propose a two-stage scheme that combines the advantages of efficient electron acceleration in a moderately low-density LWFA and efficient photon emission from energetic electrons in a relatively high-density LWFA, as shown in Fig. 1. Here, a multi-PW laser pulse is focused to a currently achievable intensity of ~10^21^ W/cm^2^, enabling it to drive a plasma bubble in a relatively high-density (~10^20^ cm^−3^) plasma according to the similarity of relativistic laser-plasma interactions (*47*) $S=\frac{n_{e}}{a_{0}n_{c}}$, where $a_{0}=\frac{eE_{y}}{m_{e}c\omega_{0}}$ is the normalized laser amplitude, *e* is the unit charge, $\omega_{0}=\frac{2\pi c}{\lambda_{0}}$ is the laser frequency, and $n_{c}=\frac{m_{e}\omega_{0}^{2}}{4\pi e^{2}}$ is the critical plasma density. In addition, a plasma channel with a transverse parabolic density profile is used to guide the high-power laser (*17*, *18*).

In the first stage, the plasma electrons are self-injected and accelerated in the plasma bubble excited by the multi-PW laser pulse propagating in an underdense plasma, resulting in a low-divergence, tens-nC, and multi-GeV electron beam with a high beam density close to the critical plasma density (10^21^ cm^−3^). The laser-to-electron energy conversion efficiency is up to ~40%. It should be pointed out that if we only consider the accelerated electrons around the quasi-monoenergetic peak, the efficiency is about 22%, consistent with the result (~20%) predicted by Gordienko and Pukhov (*47*). In the second stage, the laser pulse propagates into the relatively high-density plasma, resulting in a shrunken plasma bubble as the density increases. Besides the accelerated GeV electrons from the previous stage, additional electrons are injected in this stage, which further increases the total charge of the accelerated electron beam with a peak density well above the critical density. The efficiency increases to above 50% for the total accelerated GeV electrons as well. This results in large quasi-static electromagnetic fields around the electron beam with a radiation parameter as high as χ*_e_* ~ 0.1 defined in the following, as we can see later, which gives rise to emission of a collimated beam of γ-rays with photon energies up to the GeV level. Because the quasi-static electromagnetic fields are high enough, the radiation reaction and quantum effects begin to play an important role in the photon emission process (*33*, *37*). A distinct feature of this scheme is the high efficiency of both electron acceleration and radiation. The efficiency of multi-GeV tens-nC electrons produced is as high as 50% with an energy gain above 100 J, giving rise to an unprecedented radiation efficiency for γ-rays (with photon energy above 1 MeV) in excess of 10%, as will be shown later. Consequently, the photon number, efficiency, peak brilliance, and power of the γ-rays emitted are several orders of magnitude higher than current LWFA betatron radiation (*19*–*22*) and Compton (*23*–*25*) sources.

### Extremely bright γ-ray emission

**Fig. 2:**
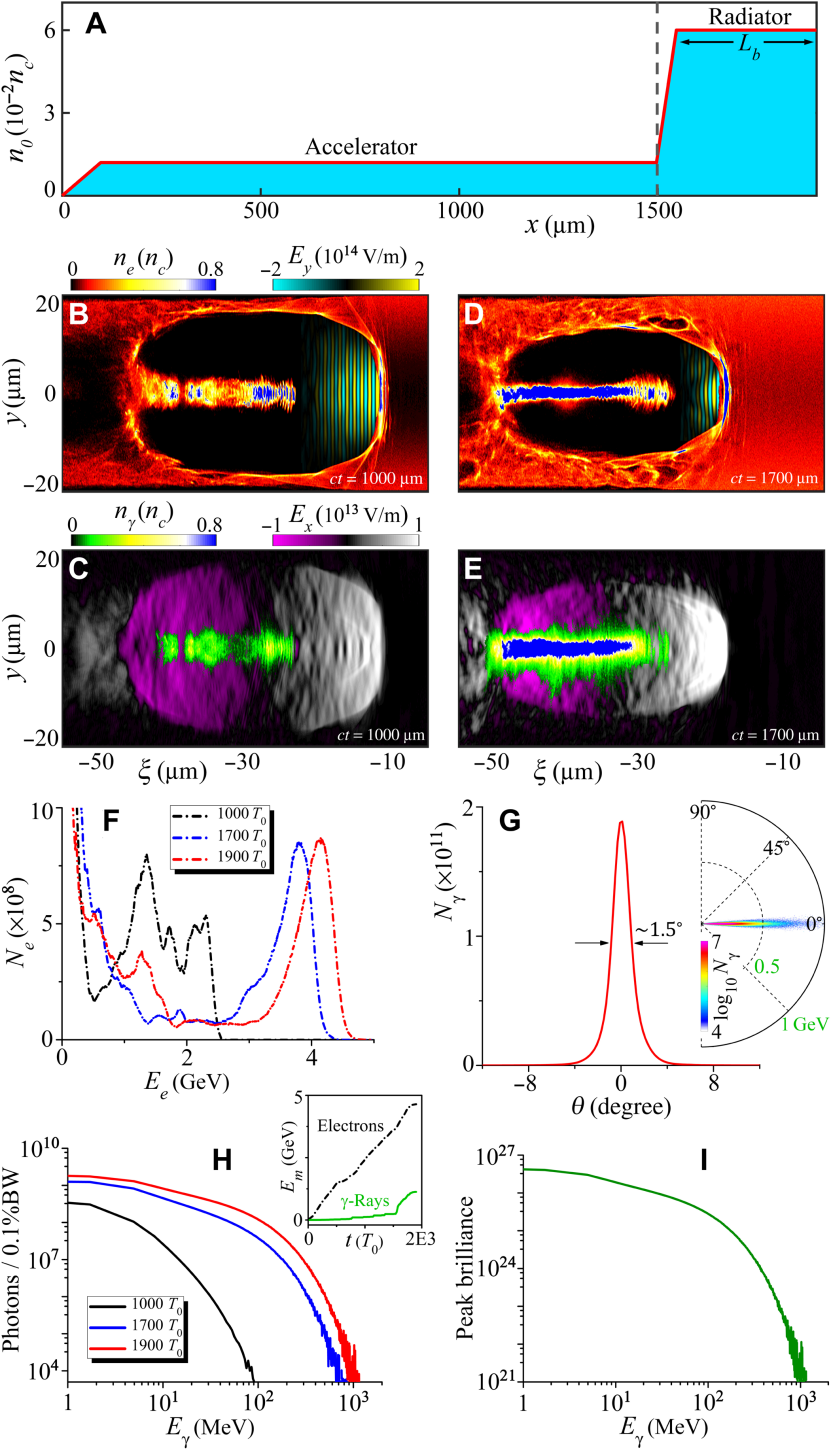
The laser-plasma accelerator-radiator setup and 3D PIC simulation results. (**A**) On-axis density profile of the background plasma. (**B** and **D**) Snapshots of distributions of the electron density (*n_e_*) and laser field (*E_y_*) are shown at time *ct* = 1000 μm and *ct* = 1700 μm, respectively, in the acceleration and radiation stages, where ξ = *x* − *ct*. Corresponding snapshots of distributions of the photon density (*n*_γ_) and accelerating field (*E_x_*) are presented in (**C**) and (**E**), respectively. The energy spectra of electrons (**F**) and γ-rays (**H**) at given times. In (H), the inset shows the temporal evolution of the maximum energy of electrons and γ-rays. (**G**) Angular spectrum and angular distribution of γ-rays. (**I**) γ-Ray peak brilliance (photons s^−1^ mm^−2^ mrad^−2^ per 0.1% BW) as a function of the emitted photon energy.

To obtain collimated ultrabright high-energy γ-ray pulses, both the charge and energy of the accelerated electron beam and the quasi-static electromagnetic fields should be high enough. To fulfill these conditions, the plasma density is longitudinally tailored to form two successive stages, one with moderately low density for efficient acceleration and the other with relatively high density for efficient radiation (see Fig. 2A).

In the acceleration stage, a plasma wake with an ultrahigh acceleration gradient above 20 GV/cm is produced by the drive laser pulse in a moderately low-density plasma channel (see Fig. 2, B and C) so that a large number of electrons are rapidly accelerated by the wake to GeV energy in millimeters. The laser pulse, together with its accelerated electron beam, then enters a higher-density plasma region in the radiator stage, where both the accelerating field ($E_{x}\propto \sqrt{n_{e}}$) and the laser fields (through relativistic self-focusing) are enhanced significantly, even though the laser depletion and electron dephasing occur faster, as shown in Fig. 2, D and E. This results in a high-density (10^21^ cm^−3^), high-charge (tens-nC), and multi-GeV electron beam (Fig. 2F). Because of high-efficiency electron acceleration occurring in high-power ultra-intense laser-plasma interactions, collimated γ-rays with photon energies up to the GeV level are copiously emitted within a narrow angular range (Fig. 2, G and H). We consider the γ-rays emitted in a 0.1% energy BW within an angle of 5 mrad, with a source size of 2 μm and a duration of 30 fs at full width at half maximum (FWHM), which gives a peak brilliance about 4 × 10^26^ photons s^−1^ mm^−2^ mrad^−2^ per 0.1% BW at 1 MeV and 1.2 × 10^26^ (usual units) at 20 MeV, as shown in Fig. 2I. One can see that the maximum peak brilliance of γ-rays may reach up to the XFEL level, making them promising high-brilliance high-energy radiation source for fundamental research and practical application.

**Fig. 3:**
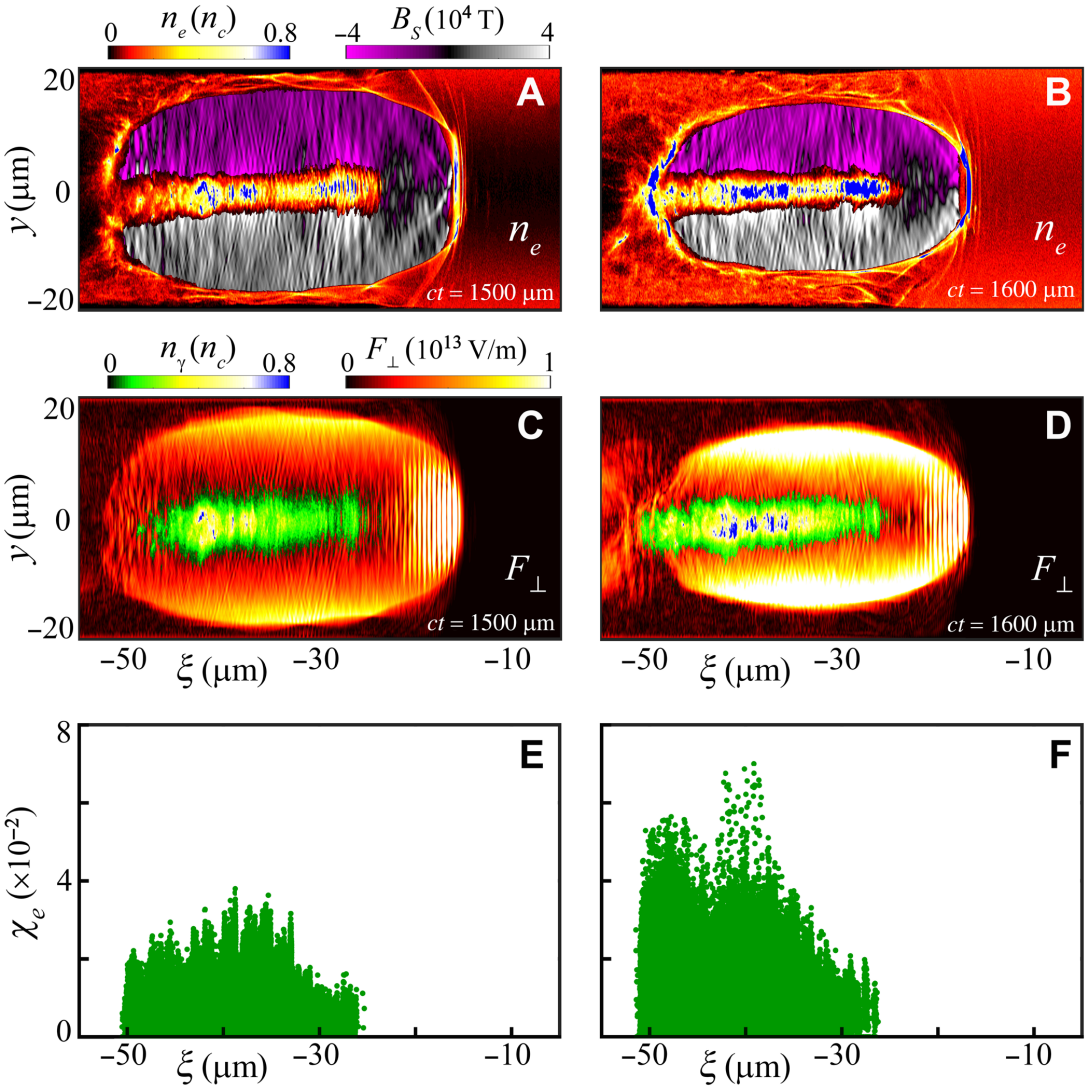
High-energy photon radiation in intense quasi-static electromagnetic fields. (**A** and **B**) Distributions of the electron density (*n_e_*) and self-generated magnetic field (*B_S_*) at *ct* = 1500 μm and *ct* = 1600 μm, respectively. (**C** and **D**) Corresponding γ-ray photon density (*n*_γ_) and transverse electromagnetic field (*F*_⊥_). (**E** and **F**) Corresponding radiation parameter (*χ_e_*) at the two positions mentioned above.

To illustrate the physical processes occurring in the two stages, we show the evolution of the plasma wake, transverse field, and radiation parameter in Fig. 3. In the acceleration stage, injection and stable acceleration of electrons are observed. The charge of trapped electrons with energy above 1 GeV is as high as 35 nC. They are accelerated to a maximum energy of ~3.6 GeV at *ct* = 1500 μm. When the laser pulse propagates into the radiator stage with a higher plasma density, the bubble shrinks as $\lambda_{w}\approx 2\pi c\sqrt{a_{0}}/\omega_{p}$, where $\omega_{p}=\sqrt{4\pi n_{e}e^{2}/m_{e}}$ is the plasma frequency. The acceleration field ($E_{x}\propto \sqrt{n_{e}}$) is simultaneously increased and has a decreased phase velocity (*17*). As a result, more electrons can be trapped and accelerated (with a charge up to ~40 nC above 1 GeV). This further enhances the self-generated fields because they scale as $2\pi en_{b}r_{b}^{2}/r$, for *r* > *r_b_* and 2π*en_b_r* for *r* ≤ *r_b_*, where *n_b_* and *r_b_* are the electron beam density and radius. The quasi-static transverse electromagnetic fields (*F*_⊥_) are composed of two parts: one component induced by the accelerated electron beam and the other associated with the plasma electron cavity (scaling as $m_{e}\omega_{p}^{2}r/2e$). As expected, with the increase of both *n_e_* and *n_b_*, *F*_⊥_ is greatly enhanced (Fig. 3D). This leads to a large radiation parameter *χ_e_* (Fig. 3F), which is proportional to *ϵ_e_* and *F*_⊥_ as $\chi_{e}\approx (\frac{e\hbar }{m_{e}^{3}c^{5}})\epsilon_{e}F_{⊥}$ (see Methods). Our simulations suggest that the radiation parameter can be as high as χ*_e_*~0.1 (see fig. S6), which is much larger than that in typical laser-irradiated solids (*39*) (χ*_e_* ≲ 0.05, producing multi-MeV photons with a very large divergence FWHM of >20^ο^), although our laser intensity is even an order of magnitude lower. Consequently, the emission of radiation by energetic electrons in a wakefield enters into the high field (strong radiation reaction) regime, in which both radiation reaction and quantum effects come into play (*48*, *49*) and copious γ-ray photons are emitted in the radiator stage.

**Fig. 4:**
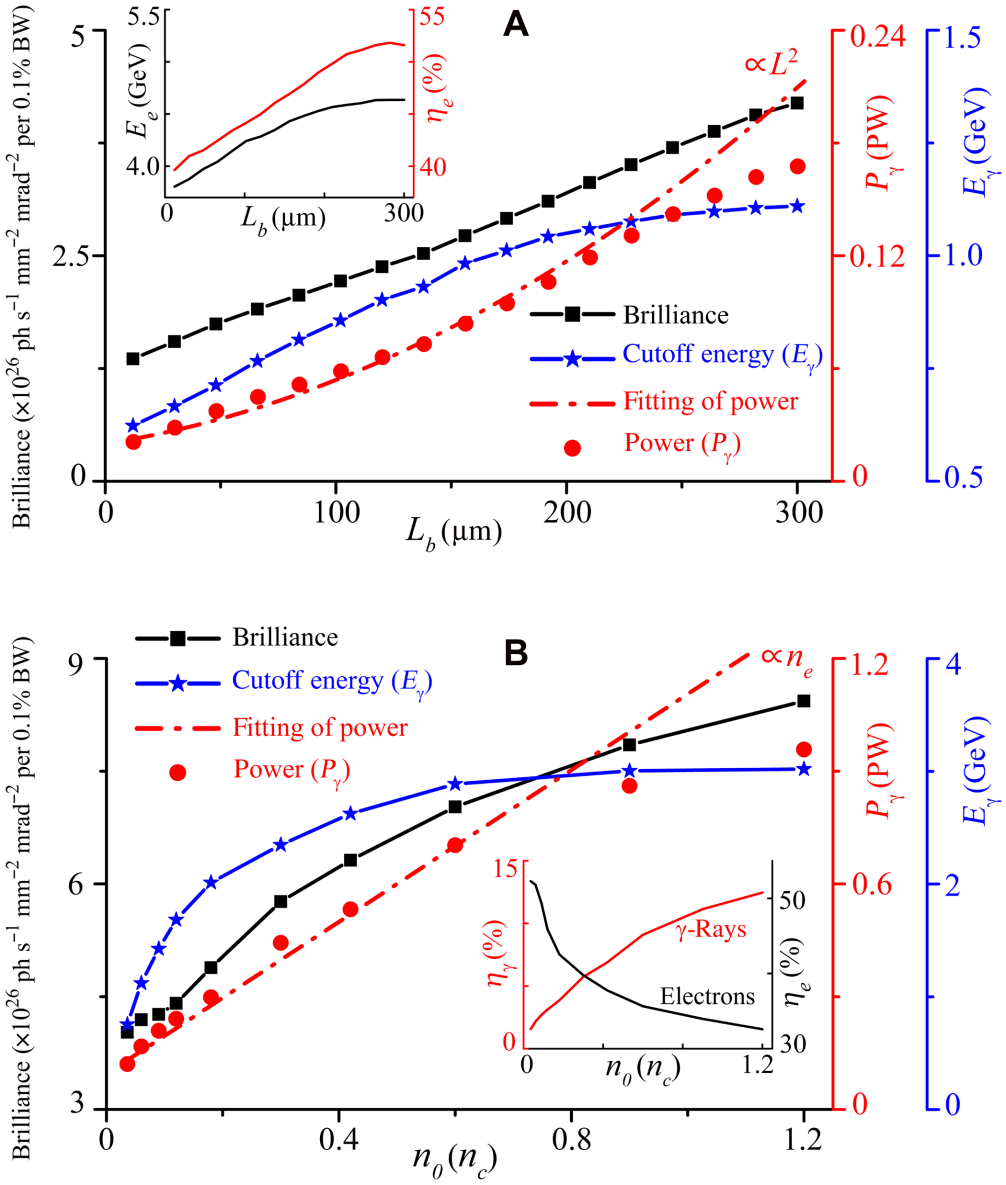
Effect of the plasma parameters on the γ-ray emission. (**A**) Effect of the radiator length (*L_b_*) on the peak brilliance at 1 MeV, cutoff energy, and radiation power of the γ-rays. The inset shows the maximum energy and total efficiency of accelerating electrons above 1 GeV. (**B**) Effect of the plasma density (*n*_0_) in the radiator region on the γ-ray peak brilliance, cutoff energy, and radiation power. The inset shows the energy conversion efficiency of trapped electrons (≥1 GeV) and γ-rays (≥1 MeV) from the drive laser. The cutoff energy of the γ-rays is defined at 10^−5^ of the peak brilliance at 1 MeV.

The maximum energy, peak brilliance, and radiation power of the emitted γ-ray can be tuned simply by changing the plasma parameters. Here, we mainly consider the effect of tuning the radiator on the γ-ray emission. The acceleration stage accelerates self-injected electrons to multi-GeV energies with a relatively low γ-ray emission level, compared with that in the radiation stage. We first discuss the effect of the radiator length *L_b_* on the γ-ray emission. Figure 4A shows the results of varying *L_b_* from 12 to 300 μm, while keeping all other parameters fixed. It shows that an appropriately long plasma radiator benefits γ-ray emission. This is due to the increased energy gain of electrons accelerated in the longer plasma segment, because ϵ*_e_* ≈ *eE_x_L*, which gives a very high efficiency (51.8%). This enables emission of copious high-energy γ-ray photons because of the substantial increase of the radiation power $P_{\gamma }\approx (\frac{2\alpha_{f}\hbar e^{2}}{3m_{e}^{4}c^{6}})\epsilon_{e}^{2}F_{⊥}^{2}$, where the power scales approximately as *P*_γ_ ∝ *L*^2^ due to the increase in *ϵ_e_*. However, the maximum radiator length and hence the maximum photon energy are limited, due to the laser pulse energy depletion and electron dephasing, e.g., for *L_b_* ≳ 250 μm.

Figure 4B illustrates the effect of plasma density on the γ-ray emission, where the product of the plasma density and length is fixed as *n*_0_*L_b_* = 18*n_c_* μm in the radiator region, and all other parameters are unchanged. The results show that γ-ray emission enhances significantly with the increase of the plasma density because $P_{\gamma }\propto \epsilon_{e}^{2}F_{⊥}^{2}\propto n_{e}$, where $\epsilon_{e}\approx eE_{x}L\propto 1/\sqrt{n_{e}}$ and $F_{⊥}\propto \frac{m_{e}\omega_{p}^{2}r}{2e}\propto n_{e}$. For example, using a density of 1.2*n_c_*, γ-ray pulses in the energy range up to 3 GeV are obtained with a peak brilliance of ~8 × 10^26^ photons s^−1^ mm^−2^ mrad^−2^ per 0.1% BW at 1 MeV and 12.5% conversion efficiency. The corresponding peak power is up to ~1 PW. This enables a substantial amount of electron energy to be efficiently transferred to high-energy photons. It should be noted, however, that the radiator density should not be too high, as it will deplete the laser pulse energy rapidly and shorten the acceleration distance (*L_b_* ∝ 1/*n*_0_). This limits the electron energy gain and thus photon emission, causing the saturation of radiation power and photon energy.

## DISCUSSION AND CONCLUSION

**Fig. 5:**
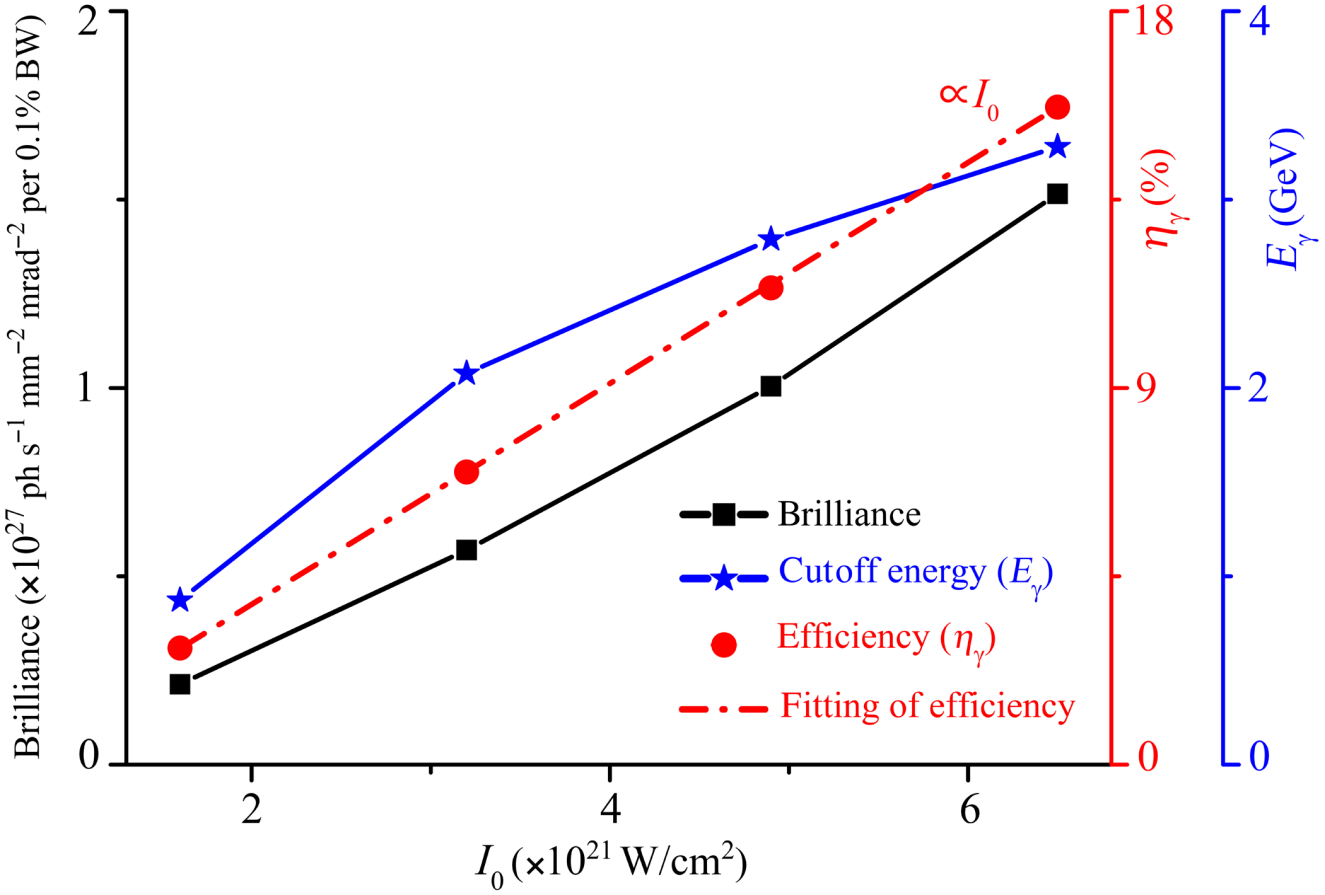
Scaling of γ-ray emission with laser intensity. Effect of the laser intensity on the peak brilliance at 1 MeV, cutoff energy, and conversion efficiency of γ-ray pulses. For high-efficiency bright γ-ray emission, the radiator plasma has a density determined by a fixed parameter *S* = *n*_0_/*a*_0_*n_c_* = 8 × 10^−3^ and length *L_b_* = 100 μm. The plasma structure, all other parameters, and corresponding *S* are the same as those shown in Fig. 2.

To demonstrate the robustness of this γ-ray radiation scheme, in Fig. 5, we have shown a series of simulations for laser intensities varied in the range 1.6 × 10^21^ to 6.5 × 10^21^ W/cm^2^. The results show that the γ-ray emission becomes more efficient as the laser intensity increases according to $P_{\gamma }\approx (\frac{2\alpha_{f}\hbar e^{2}}{3m_{e}^{4}c^{6}})\epsilon_{e}^{2}F_{⊥}^{2}\propto I_{0}^{2}$, where the electron energy scales as (*47*) $\epsilon_{e}\propto \sqrt{P_{l}}\propto \sqrt{I_{0}}$, with $P_{l}=I_{0}\pi r_{0}^{2}/2$, and the transverse field scales as $F_{⊥}\propto n_{e}\propto \sqrt{I_{0}}$ following from the relation *n_e_* = *Sa*_0_*n_c_*. Energy conversion from laser to γ-rays is defined as η = ε_γ_/ε*_l_* ∝ *P*_γ_/*P_l_*. Thus, one can obtain η ∝ *I*_0_, which agrees well with the simulation results shown in Fig. 5. As an example, using a laser pulse at the intensity 6.5 × 10^21^ W/cm^2^, the γ-ray emission can reach photon energies up to 3.3 GeV, with ~16% conversion efficiency and ultrahigh peak brilliance 1.5 × 10^27^ photons s^−1^ mm^−2^ mrad^−2^ per 0.1% BW at 1 MeV. When the laser intensity is reduced to 1.6 × 10^21^ W/cm^2^ with a peak power of 2.5 PW, bright γ-ray radiation is still quite efficient. Therefore, this scheme has the potential to operate widely in laboratories in the near future and paves the way toward a new generation of high-efficiency ultrabright GeV γ-ray sources to a broad community.

**Fig. 6:**
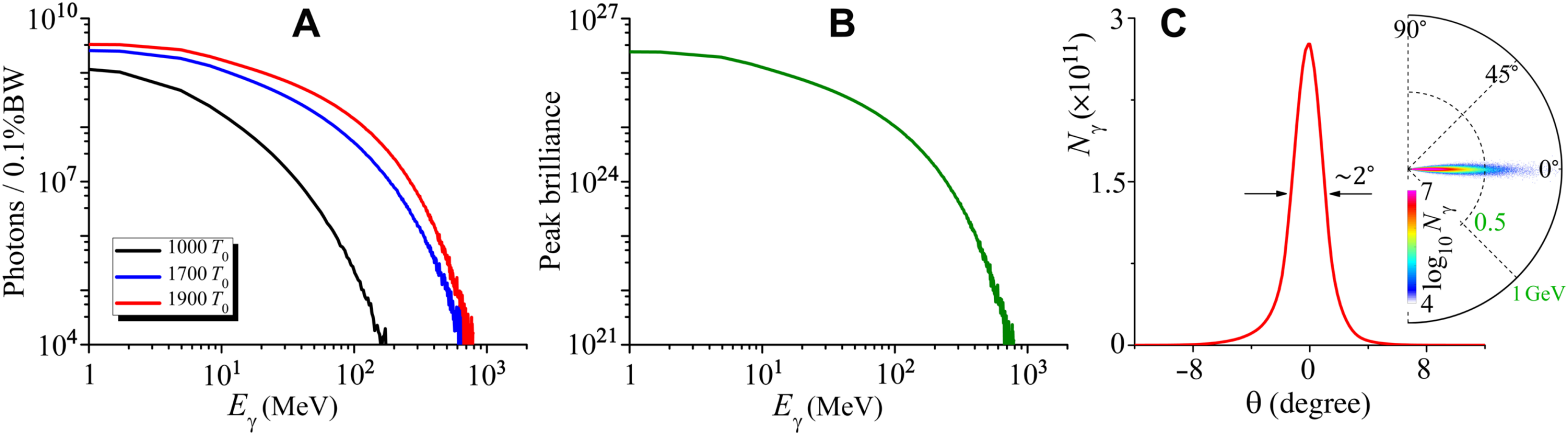
Extremely bright γ-ray source generated from a two-stage self-guided laser-plasma accelerator. (**A**) Energy spectra of the γ-ray evolution with time. (**B**) Peak brilliance (photons s^−1^ mm^−2^ mrad^−2^ per 0.1% BW) of the γ-rays emitted as a function of the photon energy. (**C**) Angular spectrum and angular distribution of the γ-rays.

We also discuss the performance of this radiation scheme in the self-guided LWFA case without the preformed plasma channel, as illustrated in Fig. 6. To extend this radiation scheme into the self-guided LWFA, the initial plasma parameters need to be adjusted accordingly, where the background density is set to *n*_0_ = 0.03*n_c_* and 0.078*n_c_* in the acceleration stage and the radiation stage, respectively. The laser parameters are the same as those presented in Fig. 2, and other parameters are unchanged but with doubled the simulation window size and corresponding grid cells. It is shown that the γ-ray radiation produced in the self-guided LWFA is still very brilliant with the peak brilliance on the order of the plasma channel case, even though the divergence angle becomes large and the emitted photon energy decreases considerably.

In summary, the previously reported X/γ-ray radiation sources from laser-driven plasma wakefields were limited to photon numbers of 10^7–8^ at hundred keV energies and GW powers, thus only delivering the pulse brilliance in the level of synchrotron light sources and radiation efficiency on the order of 10^−6^. These restrict their practical applications in broad areas. Here, we propose a novel and robust scheme capable of achieving several orders of magnitude increase in the photon number, radiation efficiency, brilliance, and power of the emitted γ-rays, based on the all-optical two-stage LWFA driven by multi-PW laser pulses. This enables the development of compact ultrashort γ-ray sources with unprecedentedly high-brilliance and PW-level powers in the GeV regime. Such powerful γ-rays may offer unique capabilities and serve as a promising new platform for various applications, such as photonuclear reactions (*5*, *6*), light-light/matter interactions (*7*, *29*–*31*), and γ-ray colliders (*4*, *50*, *51*).

## METHODS

### γ-Ray emission

It is well-known that the accelerating electrons trapped in plasma wakefields can radiate high-energy photons via betatron oscillations (*20*–*22*). When the radiated photon energy becomes comparable to the electron energy, the emission process should include the radiation reaction and quantum emission effects (*52*). In our simulations, these effects are self-consistently implemented in the particle-in-cell (PIC) code EPOCH (*53*, *54*) using a Monte Carlo method (*55*), which allows the self-consistent simulation of laser-plasma interactions in the strong electromagnetic radiation regime. The effects can be characterized by the radiation parameter

$$\chi_{e}=(e\hbar \epsilon_{e}/m_{e}^{3}c^{5})|E_{⊥}+\beta \times B|$$ (1)

Here, ϵ*_e_* is the electron energy, **E**_⊥_ is the electric field perpendicular to the electron velocity **β=v**/*c*, and $F_{⊥}=|E_{⊥}+\beta \times B|\approx \sqrt{{\left(E_{\text{sy}}-B_{\text{sz}}\right)}^{2}+{\left(E_{\text{sz}}+B_{\text{sy}}\right)}^{2}}$ is the transverse electromagnetic field that arises from the self-generated electromagnetic fields in the plasma wake. Because the trapped electrons reside at the rear of the wake and the laser pulse locates at the very front of the wake, the laser fields do not interact directly with the trapped electrons and thus do not produce the radiation by laser-electron interaction. The radiation reaction effect on electron dynamics can be expressed as $f_{\text{rr}}=-\frac{2}{3}\frac{m_{e}^{2}c^{3}}{\hbar }\alpha_{f}\chi_{e}^{2}\beta \approx -6.4\times 10^{15}\chi_{e}^{2}\beta [\text{eV}/m]$, where $\alpha_{f}=\frac{e^{2}}{\hbar c}$is the fine-structure constant. Assuming $\chi_{e}^{2}=0.001$, the radiation reaction force (∣**f***_rr_*∣ ≈ 6.4 TeV/m) becomes comparable with the electromagnetic force so that a substantial energy is transferred to high-energy photons. This strong emission has a notable impact on the electron dynamics, which should be treated using a stochastic quantum-loss model rather than using a classical continuous-loss model. Therefore, one needs to investigate the radiation process in laser-plasma interactions using self-consistent numerical simulations that include the quantum-corrected emission (*54*, *55*). The radiation power associated with radiation reaction and quantum effects can be expressed as $P_{\gamma }=(\frac{2m_{e}^{2}c^{4}\alpha_{f}}{3\hbar })\chi_{e}^{2}g(\chi_{e})$, where $g(\chi_{e})=\frac{3\sqrt{3}}{2\pi \chi_{e}^{2}}\int_{0}^{\frac{\chi_{e}}{2}}F(\chi_{e},\chi_{\gamma })d\chi_{\gamma }\approx 1/{\left(1+4.8(1+\chi_{e})\text{ln}(1+1.7\chi_{e})+2.44\chi_{e}^{2}\right)}^{2/3}$ is the correction function due to quantum effects (*55*). As *g*(χ*_e_*) ≳ 0.7 for most of the emitting electrons with χ*_e_* ≲ 0.1, one can approximately obtain

$$P_{\gamma }\approx (\frac{2\alpha_{f}\hbar e^{2}}{3m_{e}^{4}c^{6}})\epsilon_{e}^{2}F_{⊥}^{2}$$ (2)

Here, the characteristic radiated energy can be estimated as $\epsilon_{\gamma }\approx (\frac{4}{5\sqrt{3}}\frac{e\hbar }{m_{e}^{3}c^{5}})\epsilon_{e}^{2}F_{⊥}$. By assuming that the initial electron energy ϵ*_e_*~5 GeV (before emitting γ-ray photons) and that the maximum transverse field *F*_⊥_~3 × 10^13^ V/m, the maximum photon energy of the γ-rays can reach GeV level.

### Numerical simulations

Fully relativistic three-dimensional (3D) PIC simulations have been carried out by using the code EPOCH. A simulation window moving at the speed of light is used, which has absorbing boundary conditions for both fields and particles. The size of the window is 60 μm (*x*) × 45 μm (*y*) × 45 μm (*z*) with 1500 × 270 × 270 grid cells, sampled by four macroparticles in each cell. A plasma channel with a density profile of *n_e_* = *n*_0_ + *∆n*_0_ is adopted to guide the high-power high-intensity laser pulse. Here, $∆\;n_{0}=0.3a_{0}n_{c}(r^{2}\lambda_{0}^{2}/\pi^{2}r_{0}^{4})$ is the channel depth, *r* is the radial distance from the channel axis, and the on-axis background density is set to *n*_0_ = 2 × 10^−4^*a*_0_*n_c_* and 10^−3^*a*_0_*n_c_* in the acceleration and radiation stages, respectively, as shown in Fig. 2A. This type of plasma channels can be produced in several ways, such as using picosecond pulse laser–induced channeling (*15*, *56*, *57*) and laser-irradiated clusters (*58*). The incident linearly polarized laser pulse has a transversely Gaussian profile of $\text{exp}(-r^{2}/r_{0}^{2})$, a longitudinal distribution of sin^2^(π*t*/2τ_0_), and a peak intensity of $I_{0}=\frac{\pi }{2}\frac{m_{e}^{2}c^{5}}{e^{2}\lambda_{0}^{2}}a_{0}^{2}\approx 4.9\times 10^{21}\;W/{\text{cm}}^{2}$ (amplitude *a*_0_ = 60), where *r*_0_ = 10λ_0_ = 10 μm is the laser spot size and τ_0_ = 30 fs is the pulse duration (FWHM). The corresponding peak power is 7.7 PW for 232-J pulse energy. Such laser pulses are readily accessible from current multi-PW laser systems (*59*, *60*) and forthcoming laser facilities (*32*).

As a reference, we have carried out a larger-scale simulation case with the transverse dimensions of 90 μm (*y*) × 90 μm (*z*). The results are reported in the Supplementary Materials and are nearly the same as those discussed above. Furthermore, 3D PIC simulations indicate that, when the length of the plasma density up-ramp at the entrance of the radiator stage is within the range of 50 to 500 μm, it gives comparable results for the γ-ray emission. We have also carried out an additional simulation with reduced time step and cell size to investigate the effect of the numerical Cerenkov instability on the γ-ray generation, which indicates that there is no significant influence on the final results of this γ-ray radiation. The role of radiation reaction in our scheme is examined by the comparison of photon emission by energetic electrons with and without radiation reaction force, as described in the Supplementary Materials. This reveals that a self-consistently quantum-corrected radiation model must be taken into account in the emission of high-energy radiation by the electrons in extremely intense fields that we consider in this work.

## Supplementary Material

aaz7240_SM.pdf

## SUPPLEMENTARY MATERIALS

Supplementary material for this article is available at http://advances.sciencemag.org/cgi/content/full/6/22/eaaz7240/DC1

## References

[1] Corde S., Ta Phuoc K., Lambert G., Fitour R., Malka V., Rousse A., Beck A., Lefebvre E., Femtosecond X rays from laser-plasma accelerators. Rev. Mod. Phys. 85, 1–48 (2013).

[2] Bilderback D. H., Elleaume P., Weckert E., Review of third and next generation synchrotron light sources. J. Phys. B At. Mol. Opt. Phys. 38, S773–S797 (2005).

[3] Albert F., Thomas A. G. R., Applications of laser wakefield accelerator-based light sources. Plasma Phys. Control. Fusion 58, 103001 (2016).

[4] Badelek B., Blöchinger C., Blümlein J., Boos E., Brinkmann R., Burkhardt H., Bussey P., Carimalo C., Chyla J., Çiftçi A. K., Decking W., De Roeck A., Fadin V., Ferrario M., Finch A., Fraas H., Franke F., Galynskii M., Gamp A., Ginzburg I., Godbole R., Gorbunov D. S., Gounaris G., Hagiwara K., Han L., Heuer R.-D., Heusch C., Illana J., Ilyin V., Jankowski P., Jiang Y., Jikia G., Jönsson L., Kalachnikow M., Kapusta F., Klanner R., Klassen M., Kobayashi K., Kon T., Kotkin G., Krämer M., Krawczyk M., Kuang Y. P., Kuraev E., Kwiecinski J., Leenen M., Levchuk M., Ma W. F., Martyn H., Mayer T., Melles M., Miller D. J., Mtingwa S., Mühlleitner M., Muryn B., Nickles P. V., Orava R., Pancheri G., Penin A., Potylitsyn A., Poulose P., Quast T., Raimondi P., Redlin H., Richard F., Rindani S. D., Rizzo T., Saldin E., Sandner W., Schönnagel H., Schneidmiller E., Schreiber H. J., Schreiber S., Schüler K. P., Serbo V., Seryi A., Shanidze R., Da Silva W., Söldner-rembold S., Spira M., Stasto A. M., Sultansoy S., Takahashi T., Telnov V., Tkabladze A., Trines D., Undrus A., Wagner A., Walker N., Watanabe I., Wengler T., Will I., Wipf S., Yavaş Ö., Yokoya K., Yurkov M., Zarnecki A. F., Zerwas P., Zomer F., The photon collider at TESLA. Int. J. Mod. Phys. A 19, 5097–5186 (2004).

[5] Gari M., Hebach H., Photonuclear reactions at intermediate energies (40 MeV⩽Eγ⩽400 MeV). Phys. Rep. 72, 1–55 (1981).

[6] Tarbert C. M., Watts D. P., Glazier D. I., Aguar P., Ahrens J., Annand J. R. M., Arends H. J., Beck R., Bekrenev V., Boillat B., Braghieri A., Branford D., Briscoe W. J., Brudvik J., Cherepnya S., Codling R., Downie E. J., Foehl K., Grabmayr P., Gregor R., Heid E., Hornidge D., Jahn O., Kashevarov V. L., Knezevic A., Kondratiev R., Korolija M., Kotulla M., Krambrich D., Krusche B., Lang M., Lisin V., Livingston K., Lugert S., MacGregor I. J. D., Manley D. M., Martinez M., McGeorge J. C., Mekterovic D., Metag V., Nefkens B. M. K., Nikolaev A., Novotny R., Owens R. O., Pedroni P., Polonski A., Prakhov S. N., Price J. W., Rosner G., Rost M., Rostomyan T., Schadmand S., Schumann S., Sober D., Starostin A., Supek I., Thomas A., Unverzagt M., Walcher Th., Zehr F., Neutron skin of ^208^Pb from coherent pion photoproduction. Phys. Rev. Lett. 112, 242502 (2014). 24996085 10.1103/PhysRevLett.112.242502

[7] ATLAS Collaboration, Evidence for light-by-light scattering in heavy-ion collisions with the ATLAS detector at the LHC. Nat. Phys. 13, 852–858 (2017).

[8] Glinec Y., Faure J., Le Dain L., Darbon S., Hosokai T., Santos J. J., Lefebvre E., Rousseau J. P., Burgy F., Mercier B., Malka V., High-resolution γ-ray radiography produced by a laser-plasma driven electron source. Phys. Rev. Lett. 94, 025003 (2005). 15698183 10.1103/PhysRevLett.94.025003

[9] Sakdinawat A., Attwood D., Nanoscale X-ray imaging. Nat. Photon. 4, 840–848 (2010).

[10] Ackermann W., Asova G., Ayvazyan V., Azima A., Baboi N., Bähr J., Balandin V., Beutner B., Brandt A., Bolzmann A., Brinkmann R., Brovko O. I., Castellano M., Castro P., Catani L., Chiadroni E., Choroba S., Cianchi A., Costello J. T., Cubaynes D., Dardis J., Decking W., Delsim-Hashemi H., Delserieys A., Di Pirro G., Dohlus M., Düsterer S., Eckhardt A., Edwards H. T., Faatz B., Feldhaus J., Flöttmann K., Frisch J., Fröhlich L., Garvey T., Gensch U., Gerth Ch., Görler M., Golubeva N., Grabosch H.-J., Grecki M., Grimm O., Hacker K., Hahn U., Han J. H., Honkavaara K., Hott T., Hüning M., Ivanisenko Y., Jaeschke E., Jalmuzna W., Jezynski T., Kammering R., Katalev V., Kavanagh K., Kennedy E. T., Khodyachykh S., Klose K., Kocharyan V., Körfer M., Kollewe M., Koprek W., Korepanov S., Kostin D., Krassilnikov M., Kube G., Kuhlmann M., Lewis C. L. S., Lilje L., Limberg T., Lipka D., Löhl F., Luna H., Luong M., Martins M., Meyer M., Michelato P., Miltchev V., Möller W. D., Monaco L., Müller W. F. O., Napieralski O., Napoly O., Nicolosi P., Nölle D., Nuñez T., Oppelt A., Pagani C., Paparella R., Pchalek N., Pedregosa-Gutierrez J., Petersen B., Petrosyan B., Petrosyan G., Petrosyan L., Pflüger J., Plönjes E., Poletto L., Pozniak K., Prat E., Proch D., Pucyk P., Radcliffe P., Redlin H., Rehlich K., Richter M., Roehrs M., Roensch J., Romaniuk R., Ross M., Rossbach J., Rybnikov V., Sachwitz M., Saldin E. L., Sandner W., Schlarb H., Schmidt B., Schmitz M., Schmüser P., Schneider J. R., Schneidmiller E. A., Schnepp S., Schreiber S., Seidel M., Sertore D., Shabunov A. V., Simon C., Simrock S., Sombrowski E., Sorokin A. A., Spanknebel P., Spesyvtsev R., Staykov L., Steffen B., Stephan F., Stulle F., Thom H., Tiedtke K., Tischer M., Toleikis S., Treusch R., Trines D., Tsakov I., Vogel E., Weiland T., Weise H., Wellhöfer M., Wendt M., Will I., Winter A., Wittenburg K., Wurth W., Yeates P., Yurkov M. V., Zagorodnov I., Zapfe K., Operation of a free-electron laser from the extreme ultraviolet to the water window. Nat. Photon. 1, 336–342 (2007).

[11] Tajima T., Dawson J., Laser electron accelerator. Phys. Rev. Lett. 43, 267–270 (1979).

[12] Pukhov A., Meyer-ter-Vehn J., Laser wake field acceleration: The highly non-linear broken-wave regime. Appl. Phys. B 74, 355–361 (2002).

[13] Nakajima K., Fisher D., Kawakubo T., Nakanishi H., Ogata A., Kato Y., Kitagawa Y., Kodama R., Mima K., Shiraga H., Suzuki K., Yamakawa K., Zhang T., Sakawa Y., Shoji T., Nishida Y., Yugami N., Downer M., Tajima T., Observation of ultrahigh gradient electron acceleration by a self-modulated intense short laser pulse. Phys. Rev. Lett. 74, 4428–4431 (1995). 10058504 10.1103/PhysRevLett.74.4428

[14] Mangles S. P. D., Murphy C. D., Najmudin Z., Thomas A. G. R., Collier J. L., Dangor A. E., Divall E. J., Foster P. S., Gallacher J. G., Hooker C. J., Jaroszynski D. A., Langley A. J., Mori W. B., Norreys P. A., Tsung F. S., Viskup R., Walton B. R., Krushelnick K., Monoenergetic beams of relativistic electrons from intense laser–plasma interactions. Nature 431, 535–538 (2004). 15457251 10.1038/nature02939

[15] Geddes C. G. R., Toth Cs., van Tilborg J., Esarey E., Schroeder C. B., Bruhwiler D., Nieter C., Cary J., Leemans W. P., High-quality electron beams from a laser wakefield accelerator using plasma-channel guiding. Nature 431, 538–541 (2004). 15457252 10.1038/nature02900

[16] Faure J., Glinec Y., Pukhov A., Kiselev S., Gordienko S., Lefebvre E., Rousseau J.-P., Burgy F., Malka V., A laser–plasma accelerator producing monoenergetic electron beams. Nature 431, 541–544 (2004). 15457253 10.1038/nature02963

[17] Esarey E., Schroeder C. B., Leemans W. P., Physics of laser-driven plasma-based electron accelerators. Rev. Mod. Phys. 81, 1229–1285 (2009).

[18] Gonsalves A. J., Nakamura K., Daniels J., Benedetti C., Pieronek C., de Raadt T. C. H., Steinke S., Bin J. H., Bulanov S. S., van Tilborg J., Geddes C. G. R., Schroeder C. B., Tóth Cs., Esarey E., Swanson K., Fan-Chiang L., Bagdasarov G., Bobrova N., Gasilov V., Korn G., Sasorov P., Leemans W. P., Petawatt laser guiding and electron beam acceleration to 8 GeV in a laser-heated capillary discharge waveguide. Phys. Rev. Lett. 122, 084801 (2019). 30932604 10.1103/PhysRevLett.122.084801

[19] Schlenvoigt H. P., Haupt K., Debus A., Budde F., Jäckel O., Pfotenhauer S., Schwoerer H., Rohwer E., Gallacher J. G., Brunetti E., Shanks R. P., Wiggins S. M., Jaroszynski D. A., A compact synchrotron radiation source driven by a laser-plasma wakefield accelerator. Nat. Phys. 4, 130–133 (2008).

[20] Kneip S., McGuffey C., Martins J. L., Martins S. F., Bellei C., Chvykov V., Dollar F., Fonseca R., Huntington C., Kalintchenko G., Maksimchuk A., Mangles S. P. D., Matsuoka T., Nagel S. R., Palmer C. A. J., Schreiber J., Ta Phuoc K., Thomas A. G. R., Yanovsky V., Silva L. O., Krushelnick K., Najmudin Z., Bright spatially coherent synchrotron X-rays from a table-top source. Nat. Phys. 6, 980–983 (2010).

[21] Cipiccia S., Islam M. R., Ersfeld B., Shanks R. P., Brunetti E., Vieux G., Yang X., Issac R. C., Wiggins S. M., Welsh G. H., Anania M.-P., Maneuski D., Montgomery R., Smith G., Hoek M., Hamilton D. J., Lemos N. R. C., Symes D., Rajeev P. P., Shea V. O., Dias J. M., Jaroszynski D. A., Gamma-rays from harmonically resonant betatron oscillations in a plasma wake. Nat. Phys. 7, 867–871 (2011).

[22] Wenz J., Schleede S., Khrennikov K., Bech M., Thibault P., Heigoldt M., Pfeiffer F., Karsch S., Quantitative X-ray phase-contrast microtomography from a compact laser-driven betatron source. Nat. Commun. 6, 7568 (2015). 26189811 10.1038/ncomms8568 PMC4518247

[23] Ta Phuoc K., Corde S., Thaury C., Malka V., Tafzi A., Goddet J. P., Shah R. C., Sebban S., Rousse A., All-optical Compton gamma-ray source. Nat. Photon. 6, 308–311 (2012).

[24] Chen S., Powers N. D., Ghebregziabher I., Maharjan C. M., Liu C., Golovin G., Banerjee S., Zhang J., Cunningham N., Moorti A., Clarke S., Pozzi S., Umstadter D. P., MeV-energy X rays from inverse Compton scattering with laser-wakefield accelerated electrons. Phys. Rev. Lett. 110, 155003 (2013). 25167278 10.1103/PhysRevLett.110.155003

[25] Sarri G., Corvan D. J., Schumaker W., Cole J. M., Di Piazza A., Ahmed H., Harvey C., Keitel C. H., Krushelnick K., Mangles S. P. D., Najmudin Z., Symes D., Thomas A. G. R., Yeung M., Zhao Z., Zepf M., Ultrahigh brilliance multi-MeV γ-ray beams from nonlinear relativistic thomson scattering. Phys. Rev. Lett. 113, 224801 (2014). 25494074 10.1103/PhysRevLett.113.224801

[26] Holloway J. A., Norreys P. A., Thomas A. G. R., Bartolini R., Bingham R., Nydell J., Trines R. M. G. M., Walker R., Wing M., Brilliant X-rays using a two-stage plasma insertion device. Sci. Rep. 7, 3985 (2017). 28638099 10.1038/s41598-017-04124-7 PMC5479796

[27] Ferri J., Corde S., Döpp A., Lifschitz A., Doche A., Thaury C., Phuoc K. Ta., Mahieu B., Andriyash I. A., Malka V., Davoine X., High-brilliance betatron γ-ray source powered by laser-accelerated electrons. Phys. Rev. Lett. 120, 254802 (2018). 29979083 10.1103/PhysRevLett.120.254802

[28] Ta Phuoc K., Esarey E., Leurent V., Cormier-Michel E., Geddes C. G. R., Schroeder C. B., Rousse A., Leemans W. P., Betatron radiation from density tailored plasmas. Phys. Plasmas 15, 063102 (2008).

[29] Mourou G. A., Tajima T., Bulanov S. V., Optics in the relativistic regime. Rev. Mod. Phys. 78, 309–371 (2006).

[30] Marklund M., Shukla P. K., Nonlinear collective effects in photon-photon and photon-plasma interactions. Rev. Mod. Phys. 78, 591–640 (2006).

[31] Di Piazza A., Müller C., Hatsagortsyan K. Z., Keitel C. H., Extremely high-intensity laser interactions with fundamental quantum systems. Rev. Mod. Phys. 84, 1177–1228 (2012).

[32] The next generation 10 PW-level laser facilities, such as Extreme Light Infrastructure - Nuclear Physics (ELI-NP, http://www.eli-np.ro), Exawatt Center for Extreme Light Studies (XCELS, https://xcels.iapras.ru), Apollon Laser (https://apollonlaserfacility.cnrs.fr/en/home/), and Vulcan Laser (https://www.clf.stfc.ac.uk/Pages/Vulcan-2020.aspx).

[33] Zhu X.-L., Yu T.-P., Sheng Z.-M., Yin Y., Turcu I. C. E., Pukhov A., Dense GeV electron-positron pairs generated by lasers in near-critical-density plasmas. Nat. Commun. 7, 13686 (2016). 27966530 10.1038/ncomms13686 PMC5171869

[34] Zhu X.-L., Yin Y., Yu T.-P., Shao F.-Q., Ge Z.-Y., Wang W.-Q., Liu J.-J., Enhanced electron trapping and γ ray emission by ultra-intense laser irradiating a near-critical-density plasma filled gold cone. New J. Phys. 17, 053039 (2015).

[35] Huang T. W., Kim C. M., Zhou C. T., Cho M. H., Nakajima K., Ryu C. M., Ruan S. C., Nam C. H., Highly efficient laser-driven Compton gamma-ray source. New J. Phys. 21, 013008 (2019).

[36] Nakamura T., Koga J. K., Esirkepov T. Z., Kando M., Korn G., Bulanov S. V., High-power γ-ray flash generation in ultraintense laser-plasma interactions. Phys. Rev. Lett. 108, 195001 (2012). 23003049 10.1103/PhysRevLett.108.195001

[37] Ji L. L., Pukhov A., Kostyukov I. Y., Shen B. F., Akli K., Radiation-reaction trapping of electrons in extreme laser fields. Phys. Rev. Lett. 112, 145003 (2014).24765978

[38] Zhu X.-L., Yu T.-P., Chen M., Weng S.-M., Sheng Z.-M., Generation of GeV positron and γ-photon beams with controllable angular momentum by intense lasers. New J. Phys. 20, 083013 (2018).

[39] Stark D. J., Toncian T., Arefiev A. V., Enhanced multi-MeV photon emission by a laser-driven electron beam in a self-generated magnetic field. Phys. Rev. Lett. 116, 185003 (2016). 27203330 10.1103/PhysRevLett.116.185003

[40] Wang W.-M., Sheng Z.-M., Gibbon P., Chen L.-M., Li Y.-T., Zhang J., Collimated ultrabright gamma rays from electron wiggling along a petawatt laser-irradiated wire in the QED regime. Proc. Natl. Acad. Sci. U.S.A. 115, 9911–9916 (2018). 30224456 10.1073/pnas.1809649115 PMC6176611

[41] Huang T. W., Kim C. M., Zhou C. T., Ryu C. M., Nakajima K., Ruan S. C., Nam C. H., Tabletop laser-driven gamma-ray source with nanostructured double-layer target. Plasma Phys. Control. Fusion 60, 115006 (2018).

[42] Blackburn T. G., Ridgers C. P., Kirk J. G., Bell A. R., Quantum radiation reaction in laser-electron-beam collisions. Phys. Rev. Lett. 112, 015001 (2014). 24483905 10.1103/PhysRevLett.112.015001

[43] Lobet M., Davoine X., d’Humières E., Gremillet L., Generation of high-energy electron-positron pairs in the collision of a laser-accelerated electron beam with a multipetawatt laser. Phys. Rev. Accel. Beams 20, 043401 (2017).

[44] Bulanov S. S., Schroeder C. B., Esarey E., Leemans W. P., Electromagnetic cascade in high-energy electron, positron, and photon interactions with intense laser pulses. Phys. Rev. A 87, 062110 (2013).

[45] Gonoskov A., Bashinov A., Bastrakov S., Efimenko E., Ilderton A., Kim A., Marklund M., Meyerov I., Muraviev A., Sergeev A., Ultrabright GeV photon source via controlled electromagnetic cascades in laser-dipole waves. Phys. Rev. X 7, 041003 (2017).

[46] Benedetti A., Tamburini M., Keitel C. H., Giant collimated gamma-ray flashes. Nat. Photon. 12, 319–323 (2018).

[47] Gordienko S., Pukhov A., Scalings for ultrarelativistic laser plasmas and quasimonoenergetic electrons. Phys. Plasmas 12, 043109 (2005).

[48] Cole J. M., Behm K. T., Gerstmayr E., Blackburn T. G., Wood J. C., Baird C. D., Duff M. J., Harvey C., Ilderton A., Joglekar A. S., Krushelnick K., Kuschel S., Marklund M., McKenna P., Murphy C. D., Poder K., Ridgers C. P., Samarin G. M., Sarri G., Symes D. R., Thomas A. G. R., Warwick J., Zepf M., Najmudin Z., Mangles S. P. D., Experimental evidence of radiation reaction in the collision of a high-intensity laser pulse with a laser-wakefield accelerated electron beam. Phys. Rev. X 8, 011020 (2018).

[49] Poder K., Tamburini M., Sarri G., Di Piazza A., Kuschel S., Baird C. D., Behm K., Bohlen S., Cole J. M., Corvan D. J., Duff M., Gerstmayr E., Keitel C. H., Krushelnick K., Mangles S. P. D., McKenna P., Murphy C. D., Najmudin Z., Ridgers C. P., Samarin G. M., Symes D. R., Thomas A. G. R., Warwick J., Zepf M., Experimental signatures of the quantum nature of radiation reaction in the field of an ultraintense laser. Phys. Rev. X 8, 031004 (2018).

[50] Pike O. J., Mackenroth F., Hill E. G., Rose S. J., A photon-photon collider in a vacuum hohlraum. Nat. Photon. 8, 434–436 (2014).

[51] Yu J. Q., Lu H. Y., Takahashi T., Hu R. H., Gong Z., Ma W. J., Huang Y. S., Chen C. E., Yan X. Q., Creation of electron-positron pairs in photon-photon collisions driven by 10-PW laser pulses. Phys. Rev. Lett. 122, 014802 (2019). 31012720 10.1103/PhysRevLett.122.014802

[52] Ritus V. I., Quantum effects of the interaction of elementary particles with an intense electromagnetic field. J. Sov. Laser Res. 6, 497–617 (1985).

[53] Arber T. D., Bennett K., Brady C. S., Lawrence-Douglas A., Ramsay M. G., Sircombe N. J., Gillies P., Evans R. G., Schmitz H., Bell A. R., Ridgers C. P., Contemporary particle-in-cell approach to laser-plasma modelling. Plasma Phys. Control. Fusion 57, 113001 (2015).

[54] Ridgers C. P., Brady C. S., Duclous R., Kirk J. G., Bennett K., Arber T. D., Robinson A. P. L., Bell A. R., Dense electron-positron plasmas and ultraintense γ rays from laser-irradiated solids. Phys. Rev. Lett. 108, 165006 (2012). 22680729 10.1103/PhysRevLett.108.165006

[55] Ridgers C. P., Kirk J. G., Duclous R., Blackburn T. G., Brady C. S., Bennett K., Arber T. D., Bell A. R., Modelling gamma-ray photon emission and pair production in high-intensity laser–matter interactions. J. Comput. Phys. 260, 273–285 (2014).

[56] Borghesi M., MacKinnon A. J., Barringer L., Gaillard R., Gizzi L. A., Meyer C., Willi O., Pukhov A., Meyer-ter-Vehn J., Relativistic channeling of a picosecond laser pulse in a near-critical preformed plasma. Phys. Rev. Lett. 78, 879–882 (1997).

[57] Mizuta Y., Hosokai T., Masuda S., Zhidkov A., Makito K., Nakanii N., Kajino S., Nishida A., Kando M., Mori M., Kotaki H., Hayashi Y., Bulanov S. V., Kodama R., Splash plasma channels produced by picosecond laser pulses in argon gas for laser wakefield acceleration. Phys. Rev. ST Accel. Beams 15, 121301 (2012).

[58] Fukuda Y., Faenov A. Ya., Tampo M., Pikuz T. A., Nakamura T., Kando M., Hayashi Y., Yogo A., Sakaki H., Kameshima T., Pirozhkov A. S., Ogura K., Mori M., Esirkepov T. Zh., Koga J., Boldarev A. S., Gasilov V. A., Magunov A. I., Yamauchi T., Kodama R., Bolton P. R., Kato Y., Tajima T., Daido H., Bulanov S. V., Energy increase in multi-MeV ion acceleration in the interaction of a short pulse laser with a cluster-gas target. Phys. Rev. Lett. 103, 165002 (2009). 19905702 10.1103/PhysRevLett.103.165002

[59] Sung J. H., Lee H. W., Yoo J. Y., Yoon J. W., Lee C. W., Yang J. M., Son Y. J., Jang Y. H., Lee S. K., Nam C. H., 4.2 PW, 20 fs Ti:sapphire laser at 0.1 Hz. Opt. Lett. 42, 2058–2061 (2017). 28569844 10.1364/OL.42.002058

[60] Li W., Gan Z., Yu L., Wang C., Liu Y., Guo Z., Xu L., Xu M., Hang Y., Xu Y., Wang J., Huang P., Cao H., Yao B., Zhang X., Chen L., Tang Y., Li S., Liu X., Li S., He M., Yin D., Liang X., Leng Y., Li R., Xu Z., 339 J high-energy Ti:sapphire chirped-pulse amplifier for 10 PW laser facility. Opt. Lett. 43, 5681–5684 (2018). 30439927 10.1364/OL.43.005681

