# Supplementary material for: Extremely brilliant GeV γ-rays from a two-stage laser-plasma accelerator
Source: Sci Adv. 2020 May 29;6(22):eaaz7240. doi: 10.1126/sciadv.aaz7240 (PMC7259925; doi:10.1126/sciadv.aaz7240)
Supplement: aaz7240_SM.pdf [file aaz7240_SM.pdf]

Science Advances
AAAS

advances.sciencemag.org/cgi/content/full/6/22/eaaz7240/DC1

Supplementary Materials for

Extremely brilliant GeV γ-rays from a two-stage laser-plasma accelerator

Xing-Long Zhu, Min Chen*, Su-Ming Weng, Tong-Pu Yu, Wei-Min Wang, Feng He,
Zheng-Ming Sheng*, Paul McKenna, Dino A. Jaroszynski, Jie Zhang

*Corresponding author. Email: minchen@sjtu.edu.cn (M.C.); z.sheng@strath.ac.uk (Z.-M.S.)

Published 29 May 2020, *Sci. Adv.* **6**, eaaz7240 (2020)
DOI: 10.1126/sciadv.aaz7240

This PDF file includes:

Supplementary Text
Figs. S1 to S6

Supplementary Text and Figures

Effects of radiation reaction on the electron acceleration and gamma-ray generation

In order to examine the role of radiation reaction in the electron acceleration and γ-ray generation, we have carried out an additional 3D PIC simulation by switching off the radiation reaction force in the photon emission algorithm as compared to the radiation reaction case presented in Fig. 2 in the main text. In the case without radiation reaction force, the synchrotron radiation is still produced by the energetic electrons but without corresponding energy or momentum loss. The energy spectra of electrons and gamma-ray photons are shown in Fig. S1. One can see that the energy of electrons accelerated in the case without radiation reaction force is obviously higher than that produced in the case with radiation reaction force, which gives rise to larger emission rates for radiation emission and thus cause emission of higher energy photons. Therefore, for multi-PW laser driven high-energy radiation, a self-consistently quantum-corrected radiation model needs to be taken into account in order to eliminate some unreal physics.

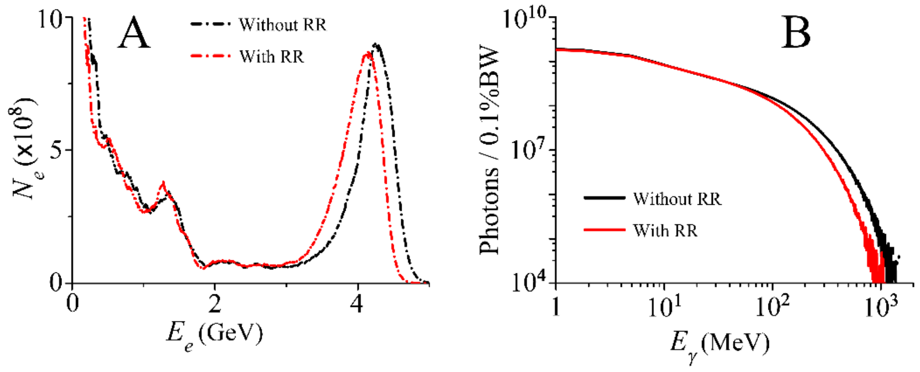

Fig. S1. Effect of radiation reaction on the energy spectra of electrons and gamma-rays. The energy spectra of accelerated electrons and emitted γ-rays from the cases with (red curve) and without (black curve) radiation reaction (RR) force by using 3D PIC simulations.

Effect of the plasma up-ramp length on the gamma-ray generation

We have investigated the effect of the length of the plasma up-ramp between the acceleration stage and the radiation stage on the γ-ray generation, as presented in Fig. S2. It is shown that, when the plasma up-ramp

length is within the range of 50 μm to 500 μm, the variation of this length has no significant influence on the peak brilliance, radiation power and photon energy of the generated γ-rays. It is noted that the plasma ramp length should not be too long, otherwise it would deplete the laser pulse significantly and thus cannot drive the radiator stage efficiently to produce higher brilliance γ-rays.

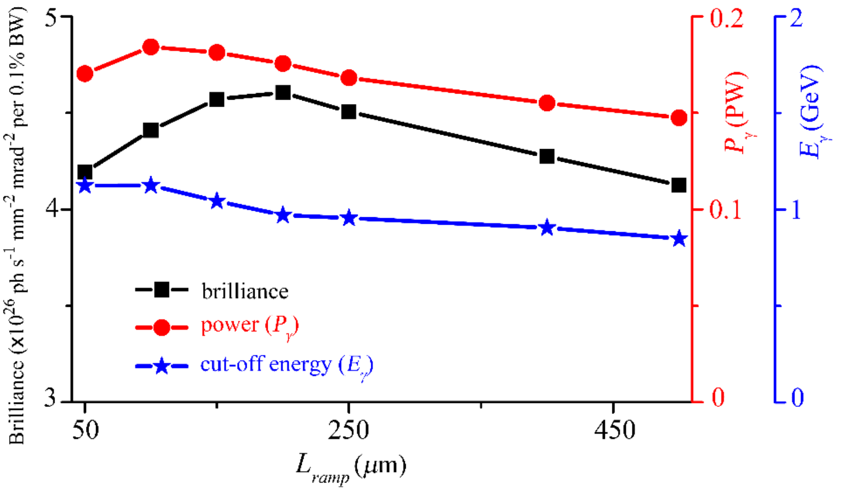

Fig. S2. Effect of the plasma up-ramp length at the entrance of the radiator stage on the γ-ray generation. The peak brilliance at 1 MeV, cut-off energy and radiation power of the γ-rays produced as a function of the plasma up-ramp length (L_{ramp}). The cut-off energy is defined at 10^{-5} of the peak brilliance at 1 MeV.

Effect of the simulation window size on the gamma-ray generation

As a reference, we have also carried out a larger-scale simulation case with transverse dimensions of $90\ \mu m\ (y) \times 90\ \mu m\ (z)$ and with other parameters unchanged, as seen in Fig. S3. It is shown that the results of the γ-ray radiation are almost the same as those reported in the main text. Therefore, the transverse dimensions of the simulation window used in our manuscript are reasonable, which have no influence on the final physical results.

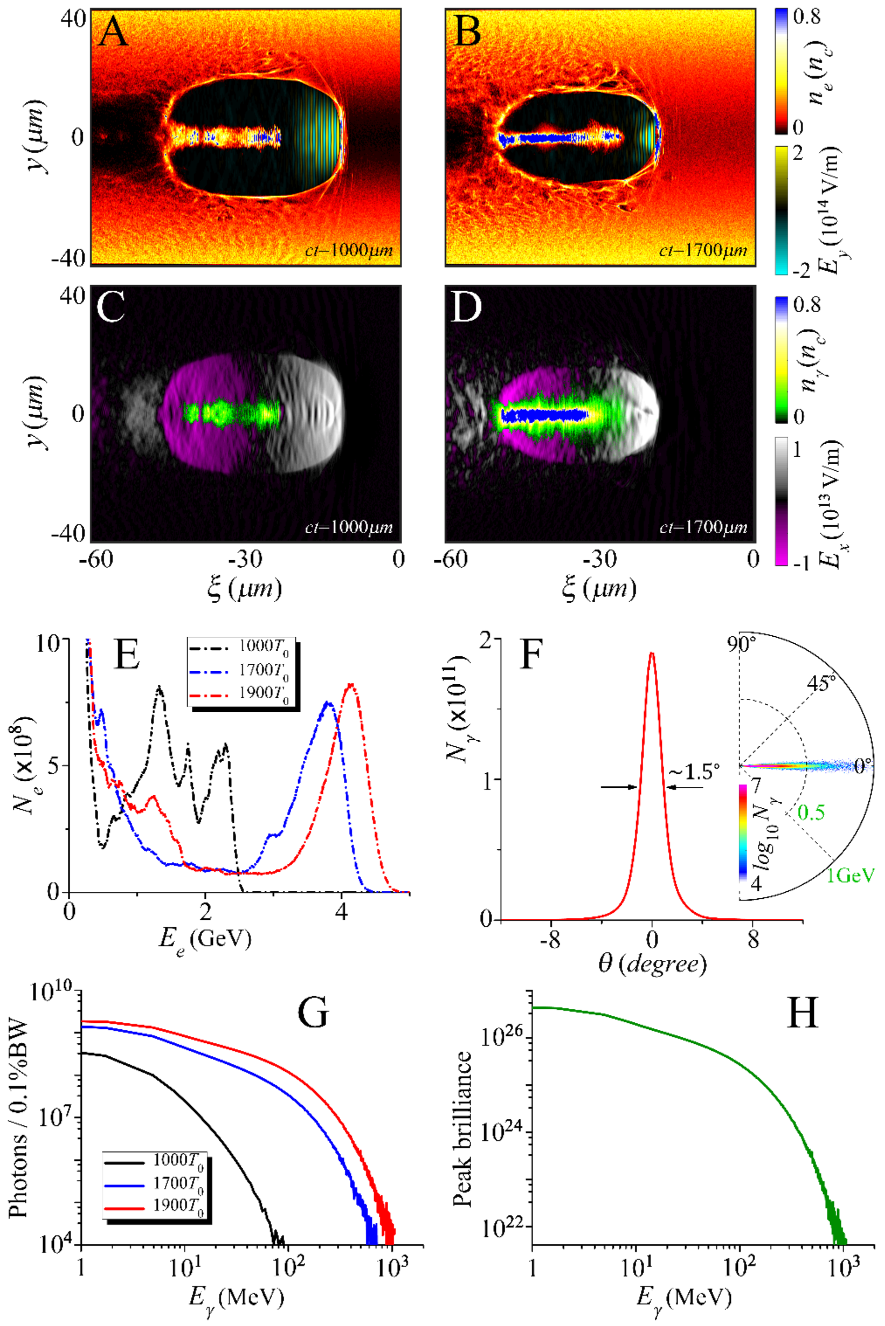

Fig. S3. Effect of the transverse dimensions of the simulation window on the γ-ray generation. (**A**, **B**) Snapshots of distributions of the electron density (n_e) and laser field (E_y) are shown at time $ct = 1000\ \mu m$ and $ct = 1700\ \mu m$, respectively, in the acceleration stage and the radiation stage, where $\xi = x - ct$. Corresponding snapshots of distributions of the photon density (n_γ) and accelerating field (E_x) are presented in (**C**) and (**D**), respectively. The energy spectra of electrons (**E**) and γ-rays (**G**) at given times. (**F**) The angular-spectrum and angular distribution of γ-rays. (**H**) The peak brilliance (photons s^{-1} mm^{-2} $mrad^{-2}$ per 0.1% BW) of the γ-rays produced as a function of the photon energy.

Effect of the numerical Cerenkov instability on the gamma-ray generation

To investigate the effect of the numerical Cerenkov instability on the γ-ray generation scheme, we have performed an additional simulation with reduced time step ($c\Delta t = 0.06\Delta x$) and cell size ($\Delta x = \lambda_0/32$), as illustrated in Fig. S4. One can see that the energy spectrum and peak brilliance of gamma-rays are nearly the same as those shown in Fig. 2 in our manuscript. Therefore, there is no significant influence on the results of this gamma-ray radiation.

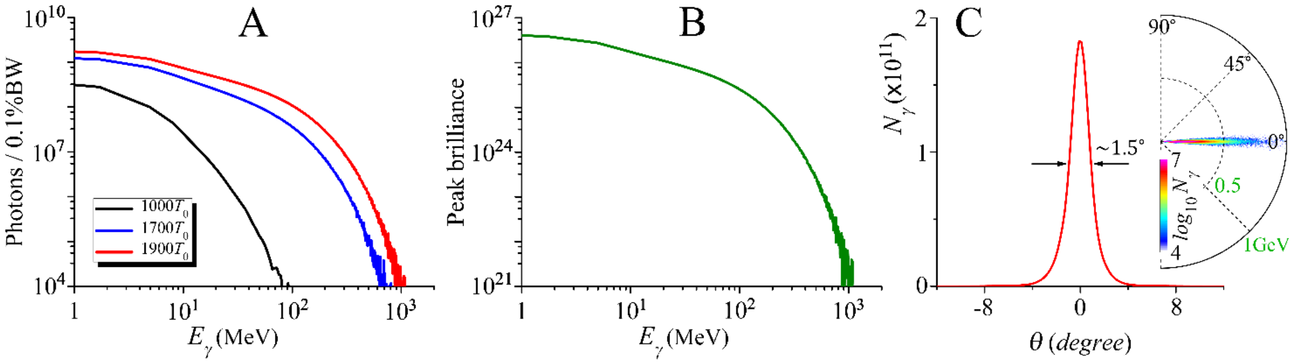

Fig. S4. Effect of the numerical Cerenkov instability on the γ-ray generation. (**A**) The energy spectra of the γ-rays emitted evolution with the time. (**B**) The peak brilliance (photons s^{-1} mm^{-2} $mrad^{-2}$ per 0.1% BW) of the γ-rays produced as a function of the photon energy. (**C**) The angular-spectrum and angular distribution of the γ-rays.

Comparison of the peak brilliance of the designed γ-ray source with other radiation sources

Figure S5 illustrates the comparison of the peak brilliance of the γ-ray radiation source generated from our two-stage scheme with other high-energy photon sources. This reveals that our scheme is a promising new way to produce collimated brilliant γ-ray flashes with peak brilliance reaching up to the XFEL level and with high energies of as high as GeV level.

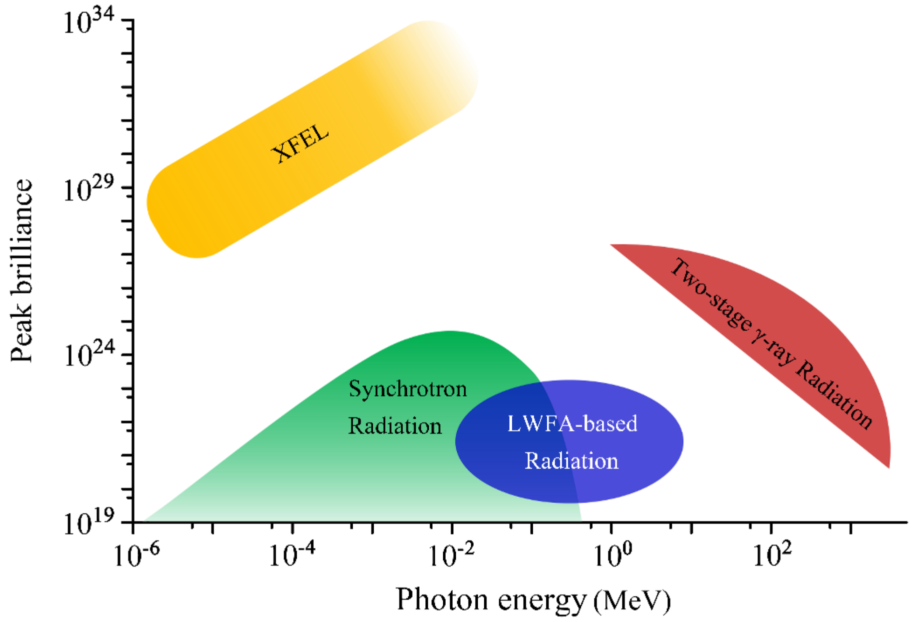

Fig. S5. The peak brilliance of the proposed γ-ray source in comparison with other radiation sources. The peak brilliance (photons s^{-1} mm^{-2} $mrad^{-2}$ per 0.1% BW) of the photon sources produced from different radiation schemes as a function of the photon energy. These peak brilliance coverages are according to the data of the references mentioned in the main text and references therein: such as see Ref. 1. Corde et al. and Ref. 3. Albert et al. for LWFA-based radiation sources; Ref. 2. Bilderback et al. for Synchrotron Radiation; and Ref. 10. Ackermann et al. for XFEL. The red region indicates the prospects of the γ-rays generated from our two-stage scheme, based-on 3D PIC simulation results.

Evolution of the self-generated magnetic field and the radiation parameter with propagation distance

Figure S6 illustrates the evolution of the self-generated magnetic field and the radiation parameter as a function of propagation distance for their corresponding maximum field. One can find that the radiation parameter increases with the self-generated magnetic field due to $\chi_e \approx \left(\frac{e\hbar}{m_e^3 c^5}\right) \epsilon_e F_\perp$, where $F_\perp$ is the transverse electromagnetic field that arises from the self-generated electromagnetic fields rather than the laser fields since the trapped electrons reside at the rear of the wake and the laser pulse locates at the very front of the wake. The self-generated magnetic field is induced mainly by the ultra-relativistic electron beam accelerated in the wake, which is enhanced significantly when the accelerated electron beam enters the high-density radiation segment, giving rise to a rapid increase of the radiation parameter as high as $\chi_e \sim 0.1$.

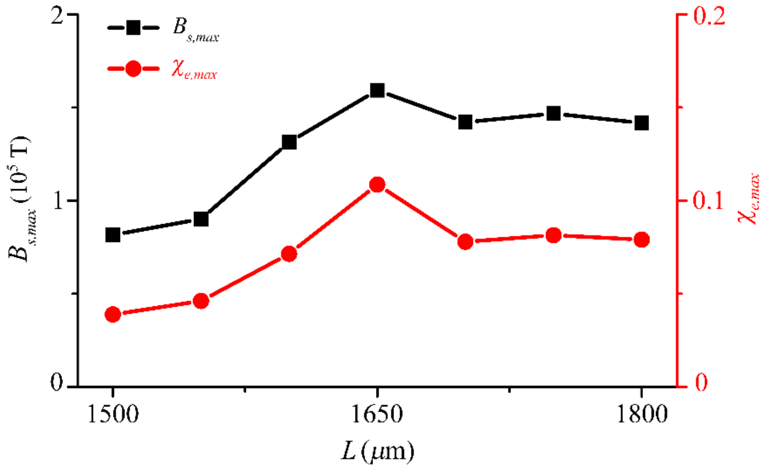

Fig. S6. Evolution of the self-generated magnetic field and the radiation parameter with propagation distance. The evolution of the self-generated magnetic field ($B_{s,max}$) and the radiation parameter ($\chi_{e,max}$) as a function of propagation distance (L) for their corresponding maximum field.
